# RETRACTION: “Aprepitant Promotes Caspase‐Dependent Apoptotic Cell Death and G2/M Arrest through PI3K/Akt/NF‐*κ*B Axis in Cancer Stem‐Like Esophageal Squamous Cell Carcinoma Spheres”

**DOI:** 10.1155/bmri/9792038

**Published:** 2026-04-01

**Authors:** BioMed Research International

RETRACTION: H. Javid, A. R. Afshari, F. Zahedi Avval, J. Asadi, and S. I. Hashemy, “Aprepitant Promotes Caspase‐Dependent Apoptotic Cell Death and G2/M Arrest through PI3K/Akt/NF‐*κ*B Axis in Cancer Stem‐Like Esophageal Squamous Cell Carcinoma Spheres,” *BioMed Research International* 2021, no. 1 (2021): 8808214, https://doi.org/10.1155/2021/8808214.

The above article, published online on 09 December 2021 in Wiley Online Library (wileyonlinelibrary.com), has been retracted by agreement between the journal Editor‐in‐Chief, Yoel A. Klug; and John Wiley & Sons Ltd. The retraction has been agreed after an investigation in figure concerns raised by *Pselaphus heisei* via PubPeer [1]. Specifically, likely duplicate western blot bands were observed between Figure 7A and Figure 4A in [2].

The Chief Editor believes that the authors’ response did not address these concerns and determined the results to be unreliable. Therefore, the article is retracted.
